# Welding Defect and Mechanical Properties of Nanosecond Laser Cleaning 6005A Aluminum Alloy

**DOI:** 10.3390/ma15217841

**Published:** 2022-11-07

**Authors:** Yuelai Zhang, Qi Yao, Weifeng Long, Chunming Wang, Ji Lin, Zehui Liu

**Affiliations:** 1 CRRC Zhuzhou Locomotive Co., Ltd., Zhuzhou 412000, China; 2 School of Materials Science and Engineering, Huazhong University of Science and Technology, Wuhan 430074, China

**Keywords:** laser cleaning, MIG welding, 6005A alloy, welding defect, mechanical properties

## Abstract

Nanosecond laser cleaning effectively removes oxide film and dirt from the surface of aluminum body parts for rail transit, as well as improving surface properties. The effect of laser cleaning on the quality of weld was studied in detail for different scanning frequencies and cleaning speeds. The effect of post-weld laser cleaning on weld quality was investigated. After laser cleaning at different parameters, the surface oxygen content was decreased and the surface roughness and surface hardness were increased. Variation of surface oxygen content was related to energy density and spot density. The lowest oxygen content was obtained at 150 W, 100 Hz and 0.8 m/min. Laser-generated craters changed surface morphology and improved surface roughness. The mechanical properties of the welded joints were slightly improved, which relates to a decrease in porosity. The minimum porosity of the laser-cleaned weld was 0.021%. This work provides new ideas for the nanosecond laser cleaning of aluminum alloy and its welding properties.

## 1. Introduction

Aluminum and aluminum alloys are widely used in rail transportation due to their high specific strength and corrosion resistance [1]. The surface of aluminum alloy easily reacts with air and forms a natural oxide film [2]. The presence of these oxide films may lead to porosity during the welding of aluminum alloys, reducing joint performance [3,4,5,6]. The aluminum oxide film causes water and oil to adsorb onto the surface, providing a source of hydrogen. It causes increased hydrogen porosity in the weld and increased porosity [7,8,9]. In order to enhance the welding quality of aluminum alloy, the oxide film must be removed from its surface before welding. The methods of removing the oxide film in the current research include chemical cleaning, mechanical polishing, electrolytic cleaning and ultrasonic cleaning [10,11,12,13]. These methods are inefficient, damaging to the health of workers, as well as increasing environmental pollution and safety risks. Therefore, an efficient and environmentally friendly method is needed to clean the oxide film on the surface of aluminum alloy.

Laser cleaning is an efficient and environmentally friendly cleaning method. In previous studies, the effect of laser cleaning on the performance and composition of aluminum alloy surfaces has been studied. The effect of laser cleaning on weld porosity and weld performance has also been investigated. T.Y. Shi studied the elemental distribution and hardness variations on the surface of aluminum alloy after laser cleaning. The surface oxygen content of aluminum alloy decreased and the surface hardness increased after laser cleaning, indicating that the surface oxide film was removed by laser cleaning. Q. Wang [14] studied the porosity distribution in the fusion zone after laser cleaning and demonstrated the feasibility of using laser cleaning to reduce the oxide layer in TIG welding of aluminum alloys. Y. Wu [15] found the strength of the aluminum alloy-bonded joint after laser treatment increased by 25%. A.W. Alshaer [16] conducted laser cleaning tests on AC170-PX aluminum sheets using a Nd:YAG pulsed laser. It was found that laser cleaning can effectively reduce porosity, as consistent with the findings of C. Zhou [17]. B. Liu [18] discussed the effect of oxygen content changes and surface morphology evolution on weld porosity after laser cleaning of 5083 aluminum alloy. The keyhole behavior reveals the micromorphology can affect the stability of the keyhole in order to regulate the porosity of the process. P. Wei [19] applied laser cleaning to the laser lap welding of SUS310S stainless steel and 6061 aluminum alloy, which effectively improved the mechanical properties of the lap joint. However, most of the research by scholars has been conducted on plates, with little direct application to aluminum alloy body parts for rail transportation. Few studies have been conducted on the welding of aluminum alloy profiles after laser cleaning.

To study the effect of laser cleaning on the welding of body parts, a nanosecond laser was used to clean the oxide film and dirt on the surface of aluminum body parts in this paper. The effect of processing on the cleaning quality and surface properties was investigated. Through MIG welding experiments, the effect of laser cleaning on the performance of lap welds and welding defects was studied. Considering the surface conditions and hard-to-clean areas, the relationship between weld defects and process parameters was analyzed. Finally, it was verified through experiments that laser cleaning after welding is not detrimental to the quality of the weld.

## 2. Materials and Methods

### 2.1. Material

The experimental materials were extruded aluminum alloy body parts for rail transportation from CRRC Zhuzhou Locomotive Co., Ltd. (6005A-T6 aluminum alloy, Zhuzhou, China). The welding wire used for manual MIG welding was SAI5087, with a diameter of 1.2 mm. The aluminum body parts were combined into lap-welded structures, which were used to study the effect of laser cleaning before and after welding on welding performance.

For laser cleaning process experiments before welding, the body parts were cut into 10 mm × 10 mm × 2 mm samples using a wire cutter. The main contaminant on the sample surface was aluminum alloy oxide film.

### 2.2. Equipment

**Figure 1:**
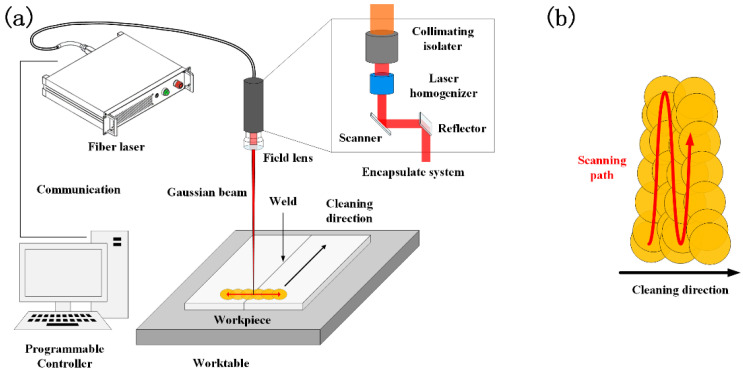
(**a**) Schematic of laser cleaning system and (**b**) the scanning path of the beam.

The laser cleaning system consisted of a fiber laser with a wavelength of 1064 nm, an encapsulate system, a robot, a worktable and auxiliary equipment shown in Figure 1a. The scanning path of the beam is shown in Figure 1b. The laser was a nanosecond laser (YDFLP-200-M7-L1-R) with a pulse width of 350 ns and a maximum average power of 200 W. The focal length of the field mirror was 254 mm. The defocusing distance used for the experiment was 0 mm. The detailed parameters of the laser were as follows: the wavelength was 1064 nm, the average power was 200 W, the pulse frequency was from 1 to 4000 kHz, the pulse width was from 8 to 500 ns, the maximum pulse energy was 1.5 mJ, the focal length was 254 mm, the spot diameter before focusing was 9 mm, the laser mode parameter was 1.8. The spot diameter after focusing was 92.9 μm.

### 2.3. Laser Processing Procedure

The surface oxygen content, surface roughness, and surface hardness were used as indicators. Single-factor experiments were conducted to study the influence of pre-weld laser cleaning process parameters (laser power, scanning frequency, cleaning speed) on the cleaning effect of aluminum alloy samples. The laser power is the average power of the laser output. The scanning frequency is the frequency of the scanner. The cleaning speed is the speed of the robot. The parameters of the laser cleaning process used for the experiments are shown in Table 1.

The body parts were cut into 15 mm × 20 mm samples for various tests. Surface oxygen content was measured by energy dispersive spectroscopy (EDS, Sirion 200, SirionLabs, Gurgaon, India). The acceleration voltage used was 20 kV, the spot was 4, and the magnification was 200×. Each sample was tested at 5 points, and the selected points were evenly distributed over the entire machined surface. The results were averaged over five points and the error bars were calculated. The composition of the chemical bonds on the surface was measured with an X-ray photoelectron spectrometer (AXIS-ULTRA DLD-600W, Kratos Analytical Ltd., Stretford, UK). The XRD spectral data was split and fitted using the CasaXPS 2.3.16 software.

Surface roughness and the top view micrograph was measured by confocal laser scanning microscope (CLSM, VK-X200K, Keyece Co., Ltd., Osaka, Japan). The magnification was 200×. Each sample was tested at nine points, and the selected points were evenly distributed over the entire machined surface. The results were averaged.

Surface hardness was measured by Vickers hardness tester (HUAYIN HVS-1000A, Laizhou Huayin Testing Instrument Co., Ltd., Yantai, China). The indenter was a quadrilateral cone with angles of 130°. The force applied was 200 g. The standard was GB/T 4340.2-1999. The test was performed at 10 points of isometry. The distance between the ten test points was 200 μm. The results were averaged. The process parameters used for the laser cleaning experiment after welding are shown in Table 2.

### 2.4. Welding Quality Assessment

Samples cleaned at different parameters were assembled using a lap-welding structure. The effect of the laser cleaning parameters on the weld properties was verified after welding. Shear strength test was performed on the welds to verify the effect of the laser cleaning parameters on the weld performance. 

Considering different surface states and hard-to-clean regions, the relationship between weld defects and process parameters was obtained by single-factor experiments. The welds were penetration tested, the penetrant testing according to ISO 3452-1 and ISO 23277-2X. The penetrant type was ZPT-5, and five-point test blocks were used. The penetration time was 15 min. Photoshop was used to extract and binarize the image of the weld area. The porosity of the processed images was counted using Image-Pro Plus 6.0 software with a tolerance of 4 pixels. The porosity Q is calculated as follows:

(1) $$Q=\frac{\sum^{\;}S_{stomata}}{S_{weld}}\times 100\%$$

where $\sum^{\;}S_{stomata}$ is the sum of all porosity area and *S_weld_* is the weld area.

The composition of black ash after welding of aluminum alloy was dominated by the high temperature oxide of impurity elements. The effect of laser cleaning after welding was verified by penetrant testing and weld performance testing. Shear strength was measured by the universal testing machine (Instron 8801). The welding method used in the experiment was manual MIG welding with a shielding gas of 99.99% argon gas. For the MIG welding process, the arc voltage was 23.2 V, the welding current was 175 A, and the gas flow rate was 22 L/min.

## 3. Results

### 3.1. Surface Oxygen Content

**Figure 2:**
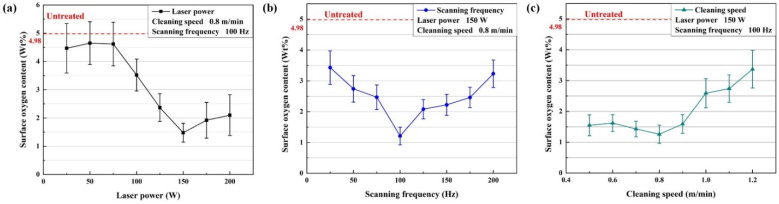
The variation of the surface oxygen content with the process parameters: (**a**) laser power; (**b**) scanning frequency; (**c**) cleaning speed.

The surface oxygen content after laser cleaning directly reflects the residual oxide film on the surface of the aluminum alloy. Figure 2 shows the variation of the surface oxygen content of the aluminum alloy with the process parameters. As shown in Figure 2a, the surface oxygen content first decreased and then increased with increasing laser power. The lowest surface oxygen content was reached at a laser power of 150 W, indicating that the aluminum alloy sheet had been basically cleaned of the oxide film. In Figure 2b, the trend of the oxygen content on the surface with increasing scanning frequency is consistent with the variation in laser power. At 100 Hz, the surface oxygen content reached the lowest. Figure 2c shows the surface oxygen content first decreased and then increased as the cleaning speed increased. At 0.8 m/min, the surface oxygen content reached the lowest. The error of the surface oxygen content after laser cleaning decreased as the oxygen content decreased. Error bar of the surface oxygen content is minimized at the optimal parameter.

**Figure 3:**
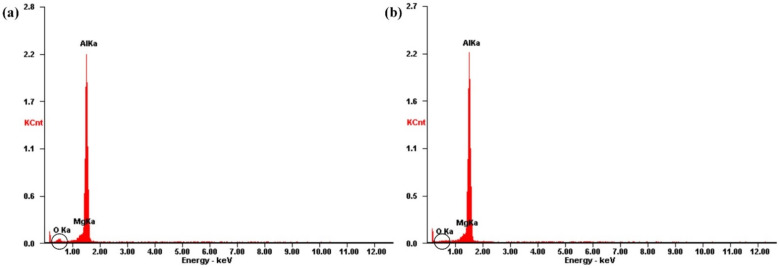
The results of EDS on the surface of samples: (**a**) untreated; (**b**) laser-cleaned.

At 150 W, 100 Hz, and 0.8 m/min, the results of EDS on the surface of the untreated sample and the laser-cleaned sample are shown in Figure 3. The EDS result of untreated samples showed a peak of oxygen element. After laser cleaning, the peak of oxygen element dropped to almost disappear. It confirms that the surface oxygen content decreased after laser cleaning.

**Figure 4:**
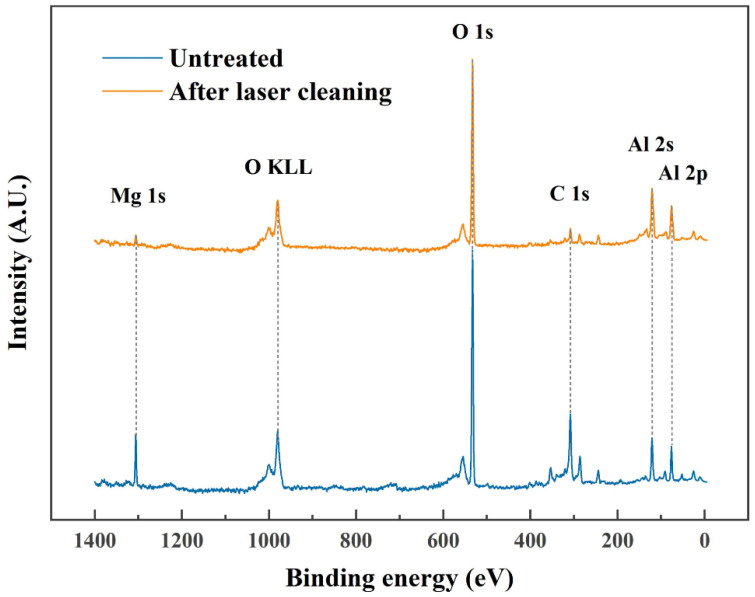
The XPS spectrum of the surface of 6005A alloy with untreated and laser cleaned.

The full spectrum of XPS was used to study the composition of oxide films before and after laser cleaning. Figure 4 shows that the surface oxides before and after laser cleaning are mainly alumina and a small amount of magnesium oxide. The intensity of Mg 1s was reduced after laser cleaning, which means that laser cleaning removes most of the magnesium oxide.

**Figure 5:**
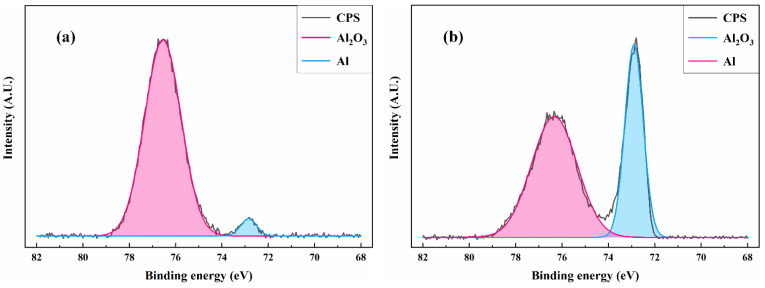
The XPS spectrum of Al 2p: (**a**) untreated; (**b**) laser cleaning.

To study the changes of surface alumina, the detailed XPS spectrum of Al 2p was analyzed. As shown in Figure 5, the composition of the untreated surface was mainly Al_2_O_3_ and a few Al. The composition of the surface material was not changed after laser cleaning. The signal intensity of Al increased while that of Al_2_O_3_ decreased, it means that laser cleaning removes alumina from the surface.

### 3.2. Surface Roughness

**Figure 6:**
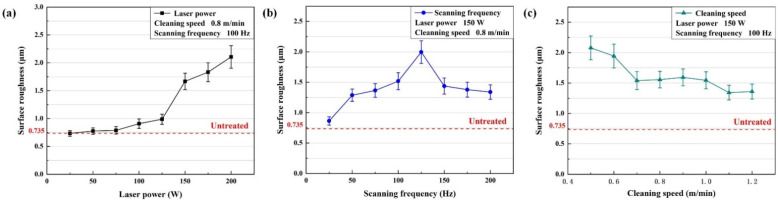
The variation of the surface roughness with the process parameters: (**a**) laser power; (**b**) scanning frequency; (**c**) cleaning speed.

Laser cleaning has an effect on the surface roughness (Ra) of the sample. The surface roughness variation is shown in Figure 6. After laser cleaning, the surface roughness of the sample increased. As shown in Figure 6a, the surface roughness increased as laser power increased. At 200 W, the surface maximum roughness was 2.106 μm. Figure 6b shows the surface roughness increased and then decreased with the increase in scanning frequency. At 125 Hz, the maximum surface roughness was 1.996 μm. The change of surface roughness with the cleaning speed is shown in Figure 6c. The roughness basically decreased with the increase of cleaning speed.

### 3.3. Surface Hardness

**Figure 7:**
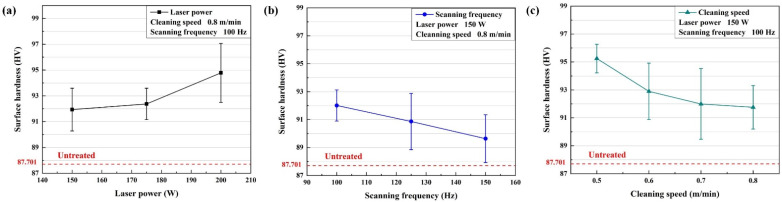
The variation of the surface hardness with the process parameters: (**a**) laser power; (**b**) scanning frequency; (**c**) cleaning speed.

The surface hardness of the aluminum alloy slightly increased after laser cleaning. The surface hardness variation is shown in Figure 7. The range of parameters of the process was narrowed according to the oxygen content and roughness. Mainly, the laser power was 150 W–200 W, the scanning frequency was 100 Hz–150 Hz, and the cleaning speed was 0.5 m/min–0.8 m/min.

As shown in Figure 7a,b, the hardness of the cleaned surface rose with the laser power at 150 W–200 W. The maximum hardness was 94.782 HV. At 100–150 Hz, the hardness of the cleaned surface decreased as the scanning frequency rose. The maximum hardness was 92.011 HV at 100 Hz. Figure 7c shows that as the cleaning speed increased, the hardness of the cleaned surface also decreased. At 0.5 m/min, the surface hardness was up to 95.245 HV. Compared to untreated samples, the surface hardness of the cleaned samples increased by up to 8.601%.

**Figure 8:**
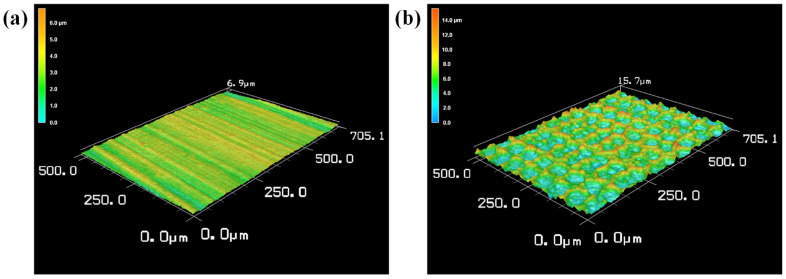
The top view micrograph of laser-cleaned and untreated surfaces: (**a**) untreated; (**b**) laser-cleaned.

At 150 W, 100 Hz, and 0.8 m/min, the top view micrograph of laser-cleaned and untreated surfaces are shown in Figure 8. Scratches were clearly observed on the untreated surface. After laser cleaning, the scratches were removed. The craters were observed on the surface of the sample after laser cleaning, the overlap of the craters was good. 

### 3.4. Weld Performance

**Figure 9:**
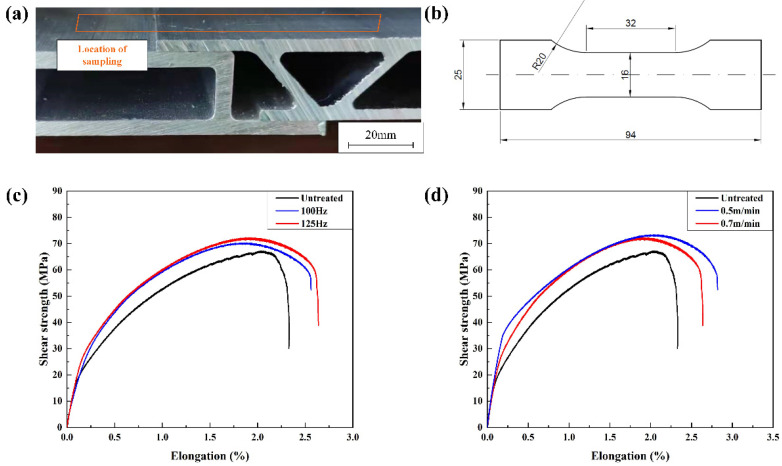
(**a**) The weld structure; (**b**) the dimension of the shear specimen and the variation of the shear strength with the process parameters: (**c**) scanning frequency; (**d**) cleaning speed.

There was a height difference in the aluminum body parts used in the experiment. To ensure the quality of the laser cleaning, the laser power was fixed at 200 W. The weld structure and the dimension of the shear specimen are shown in Figure 9a,b. The variation of weld shear strength with process parameters are shown in Figure 9c,d.

The laser-cleaned samples had slightly higher weld shear strength and elongation than the untreated samples. As shown in Figure 9a, the shear strength and elongation of the weld increased slightly as the scanning frequency increased. As shown in Figure 9b, the shear strength and elongation of the weld increased slightly as the cleaning speed decreased. Laser cleaning process parameters could be adjusted to slightly improve the mechanical property of the weld.

### 3.5. Welding Defect

**Figure 10:**
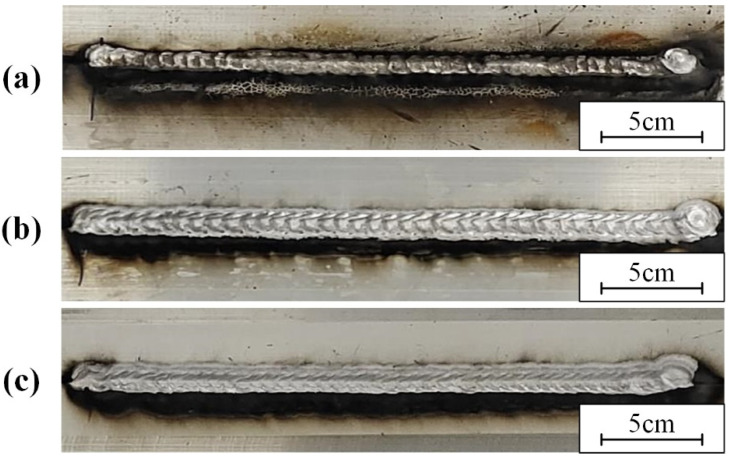
The weld formation on sample with: (**a**) oil; (**b**) water; (**c**) laser cleaning.

**Figure 11:**
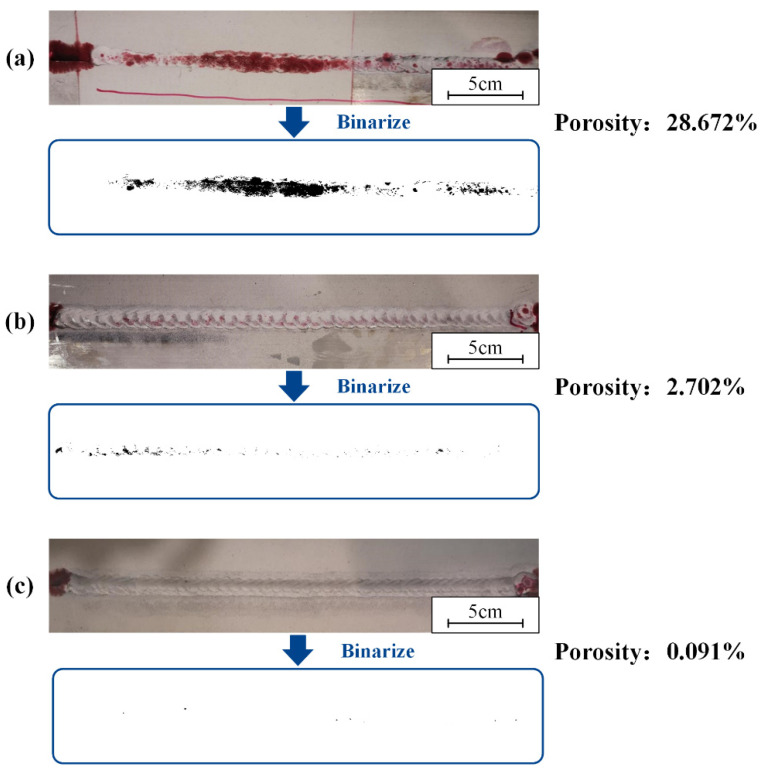
The result of the permeation testing and the porosity at sample with: (**a**) oil; (**b**) water; (**c**) laser cleaning.

A cleaning speed of 0.7 m/min, a scanning frequency of 100 Hz, and a laser power of 200 W were used in this part. Different surface states had an effect on the forming quality of the weld. Welding was performed on samples with oil and water stains on the surface. The weld of the laser-cleaned sample was compared with them. The weld formation is shown in Figure 10. The result of the penetrant testing and the surface porosity at different surface states are shown in Figure 11. Defects caused by arc suppression and arc starting are not considered in the experiment.

The oil pollution will affect weld formation. As shown in Figure 10a, the samples with oil had unstable weld pools and more black ash after welding. A large number of porosities and weld inclusions are shown in Figure 11a. Figure 10 shows that both the sample with water and the laser-cleaned sample have well-formed welds. Figure 11b shows the sample with water has a row of small porosities in the weld. As shown in Figure 11c, the weld of the laser-cleaned sample is basically free of porosity.

**Figure 12:**
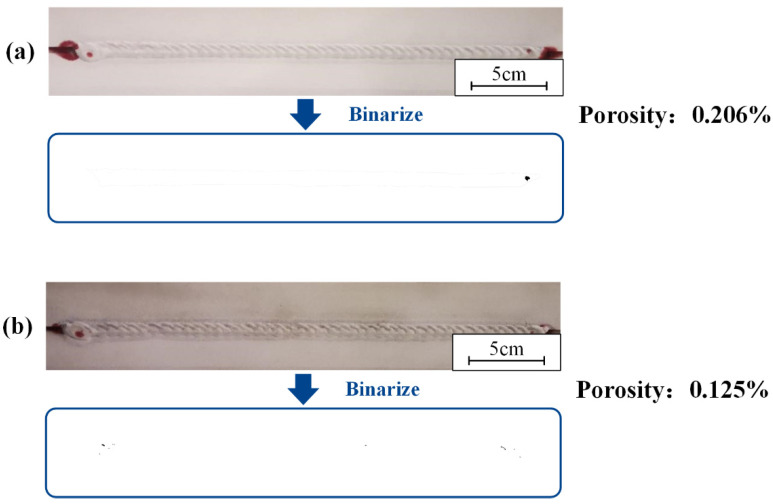
The result of the permeation testing and the porosity: (**a**) the lap surface was not cleaned; (**b**) the lap surface was cleaned.

The effect of cleaning or not cleaning the lap surfaces on the weld formation was studied. The results of weld penetration testing and the porosity with and without laser cleaning within the lap surface are shown in Figure 12. The welds were studied. There was basically no defect in the middle of the weld, and the quality of the weld was qualified. The porosity of the samples after laser cleaning of the lap surface was slightly lower than that of the samples without cleaning of the lap surface.

### 3.6. Correlation of Process Parameters with Welding Defects

**Figure 13:**
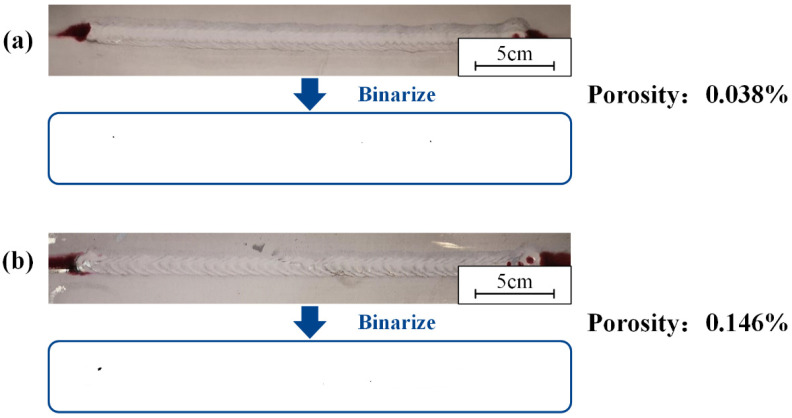
The result of the permeation testing and the porosity under different scanning frequencies: (**a**) 100 Hz; (**b**) 125 Hz.

The laser-cleaning process fixed the laser power at 200 W. The scanning frequencies were set to 100 Hz and 125 Hz. Cleaning speeds were set to 0.5 m/min and 0.7 m/min, respectively. For the welds, the results of weld penetrant testing and porosity at different scanning frequencies are shown in Figure 13. The cleaning speed was 0.7 m/min. As shown in Figure 13, the welds were almost free of defects. The porosity increased slightly with increasing scan frequency. At 125 Hz, the porosity was 0.146%, an improvement of 0.108% compared to 0.5 m/min. The quality of the welds at both 100 Hz and 125 Hz was qualified. The results of weld penetrant testing and porosity at different cleaning speeds are shown in Figure 13. The scanning frequency was 125 Hz.

**Figure 14:**
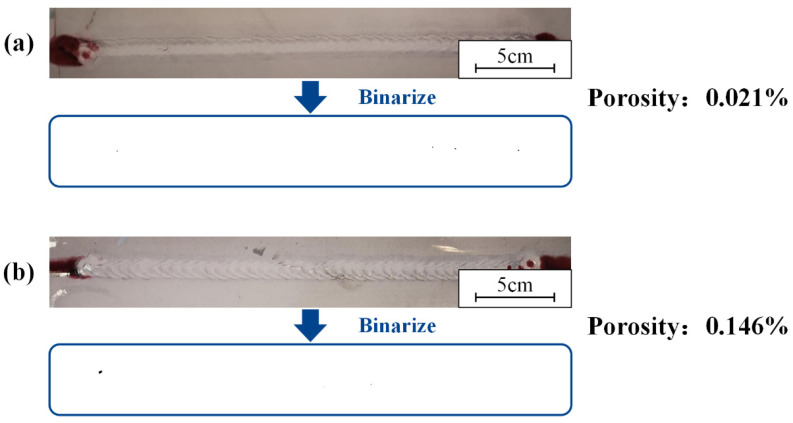
The result of the permeation testing and the porosity under different cleaning speeds: (**a**) 0.5 m/min; (**b**) 0.7 m/min.

As shown in Figure 14, there were almost no defects in the weld. The porosity decreased slightly with increasing cleaning speed. At 0.7 m/min, the porosity was 0.146%, an improvement of 0.125% compared to 0.5 m/min. The quality of the welds at both 0.5 m/min and 0.7 m/min were qualified.

### 3.7. Weld Performance and Defect of Post-Weld Cleaning

**Figure 15:**
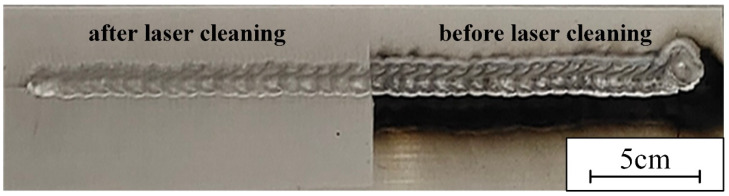
The weld formation before and after laser cleaning of black ash.

**Figure 16:**
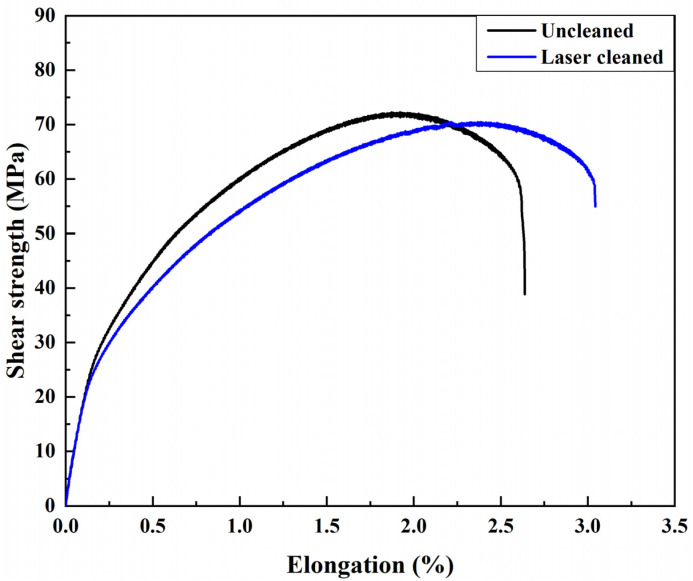
The shear strength of the samples before and after black ash removal.

Black ash after welding was laser-cleaned. For the weld, the black ash of the weld in the cleaning area was completely removed, and there were no obvious defects in the weld. The weld formation is shown in Figure 15. The shear strength of the samples before and after black ash removal are shown in Figure 16.

**Figure 17:**
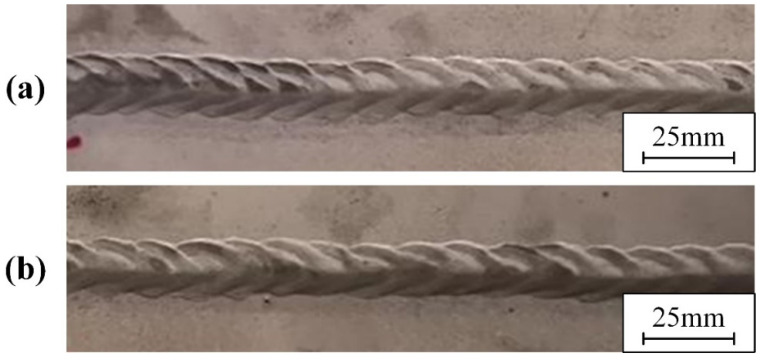
The result of the permeation testing: (**a**) black ash without laser cleaning; (**b**) black ash with laser cleaning.

Figure 16 shows that laser cleaning of the black ash was not detrimental to the weld shear strength. The weld elongation was slightly improved. Laser cleaning of black ash had a slight strengthening effect on the weld performance. The weld composed of the air-conditioning plate and the side beam segment were used to test the weld defect. The penetrant testing results before and after laser cleaning of black ash are shown in Figure 17. The welds were well-formed and essentially free of defects. Laser removal of surface black ash had no effect on the results of the penetration test. No new defects were produced by laser cleaning after welding.

## 4. Discussion

The surface oxygen content first decreases and then increases with increasing laser power. B. Liu [18] and C.N. Afonso [20] have shown the laser energy density can adjust the oxygen content. Below the threshold for oxidative damage, the oxygen content decreases with increasing energy density. After the energy density exceeds the oxidative damage threshold, thermal oxidation occurs. Laser cleaning forms craters on the surface of aluminum alloys. The peaks and troughs of the cross-section are formed because of the craters. G. Zhang [21] found that the surface roughness increased due to the craters. B. Liu [18] found that a laser-irradiated aluminum alloy surface formed a molten pool. The depression in the center of the melt pool is caused by the backlash pressure. The molten pool solidifies rapidly under quenching of the low-temperature substrate. The surface tension and gravity lead to the formation of crater morphology.

As shown in Figure 2a, the lowest oxygen content was obtained at 150 W. The energy density is calculated as follows [22]:

(2) $$E_{0}=\frac{4P}{\pi D^{2}F_{p}}$$

(3) $$D=4\lambda fM^{2}/\pi d$$

where *E*_0_ is the energy density; *P* is the laser power; *D* is the spot diameter after focusing; *F_p_* is the pulse frequency; *λ* is the wavelength; *f* is the focal length; *M*^2^ is the laser mode parameter; *d* is the spot diameter before focusing. By Equations (2) and (3), with 150 W, the energy density is 17.02 J/cm^2^. The surface temperature rise of Al_2_O_3_ under laser is calculated as follows [18]:

(4) $$\Delta T=2E_{0}\beta \sqrt{\alpha \tau }/\left(\lambda_{t}\tau \sqrt{\pi }\right)$$

where Δ*T* is the surface temperature rise; *β* = 1 − R and R is the reflectivity; *α* is the thermal diffusion coefficient; *λ_t_* is the thermal conductivity; *τ* is the pulse width.

**Figure 18:**
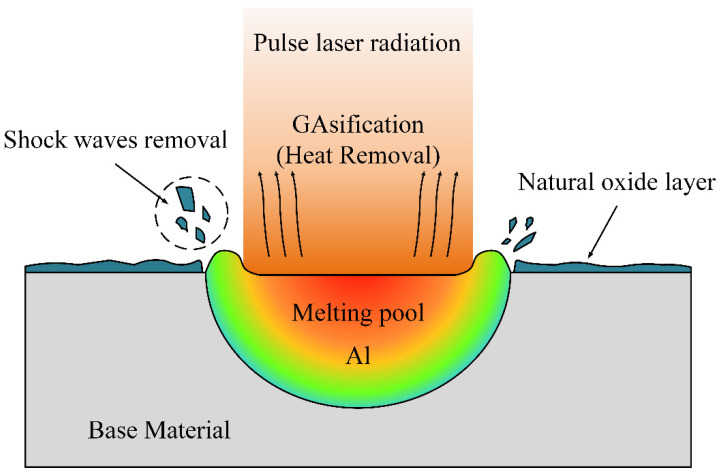
The schematic of laser cleaning principle.

In previous studies, the reflectivity of Al_2_O_3_ to 1064 nm laser is about 5.6 [18]. The thermal conductivity is 33 W/(m⋅K). The thermal diffusion coefficient is 0.111 cm^2^/s [23]. The boiling point of Al_2_O_3_ is 2980 °C [24]. By Equation (4), the minimum power to reach the boiling point of the oxide film is 57.3 W. At 75 W–150 W, the oxide is removed under the action of shock waves [25,26,27,28] or heat [27,29]. The principle of laser cleaning is shown in Figure 18. Laser cleaning after the oxide film composition had not changed as shown in Figure 8. With the increase in laser power, the energy density increased, the boiling efficiency of the oxide film was increased. The oxide removal mechanism transformed from heat removal to the combination of heat removal and shock wave removal as the energy density rose. Oxide film removal increased with power. The power continued to increase, the high energy density leads to thermal oxidation. However, compared to untreated surfaces, the oxygen content was still low at 200 W. As shown in Figure 6a, the increase in power caused the crater depth to increase, resulting in an increase in surface roughness.

The cleaning effect is related to the spot density [17]. The higher the scanning frequency, the faster the scanning speed of the beam, but the smaller the cleaning distance in a cycle. As the scan frequency increases, the spot density on the scan path decreases, and the spot density on the cleaning direction increases. Low spot density can lead to poor cleaning results. As shown in Figure 2b, at 100 Hz, the oxygen content was the lowest. Less than 100 Hz, the spot density on the cleaning direction was not high enough. More than 100 Hz, the spot density on the scan path was not high enough. The spot density in the cleaning direction is inversely proportional to the cleaning speed. Figure 2c shows that at less than 0.8 m/min, the oxygen content was low. The spot density determines the number of craters. Figure 6b shows the volcanic crater was most evenly distributed on the surface at 125 Hz. As the cleaning speed increases, the number of craters in the cleaning direction decreases. As shown in Figure 6c, the surface morphology was closer to that of untreated samples at 1.2 m/min. The spot density determines the number of craters. Figure 6b shows the volcanic crater was most evenly distributed on the surface at 125 Hz. As the cleaning speed increases, the number of craters in the cleaning direction decreases. As shown in Figure 6c, the surface morphology was closer to that of untreated samples at 1.2 m/min.

The melt pools re-solidify to form a hardened layer [30]. M. Moshtaghi’s study [31] shows that different heat input and cooling rates lead to the formation of different microstructures of the material surface. More small-angle grain boundaries are formed during re-solidification, the re-solidified layer has a greater dislocation density. As the laser power increases, the energy density increases leading to an increase in heat input. The short pulse width allows a high cooling rate to be maintained even when the energy density increases, so that the grain size of the hardened layer is less varied at different powers [32]. Increased melting of the material surface leads to deeper melt pool depths. The hardened layer thickens after solidification and the surface hardness increases. Higher spot density leads to similar results. Therefore, the surface hardness decreases with the increase in cleaning speed. As shown in Figure 2, Figure 13 and Figure 14, after laser cleaning, the porosity increased with the increase of the surface oxygen content. After laser cleaning, the surface oxygen content of the sample was reduced causing porosity to decrease. The research of A.W. AlShaer [16] also confirmed this conclusion. The oxide film reacts thermally during the welding process to produce gas. The gas does not escape in time to cause porosity. The weld performance is worse due to higher porosity [3,5]. When there are porosities in the welded joint, the fracture starts near the porosity. Lower porosity leads to higher shear strength. The weld shear strength increased due to the reduced porosity after laser cleaning, as shown in Figure 9. As the degree of laser cleaning increased, lower porosity was obtained as shown in Figure 10 and Figure 14. As a result, the shear strength of the cleaned specimen increased. The oil causes difficulty in the arc starting, and the welding process is unstable. So as shown in Figure 10, the oil makes the weld formation worse. The hydrogen element in the oil decomposes directly at high temperatures to form H_2_ [9,16]. Hydrogen readily dissolves in high temperature liquid aluminum. The cooling and solidification of the melt pool causes the hydrogen to precipitate and produce bubbles. When the crystallization speed is higher than the bubble spillage speed, hydrogen pores are formed [18]. Therefore, a large number of porosities and weld inclusions are shown in Figure 11a. Liquid aluminum reacts with water to produce H [16], the hydrogen element in water leads to the creation of hydrogen porosity. Figure 11b shows the sample with water had few small-size porosities in the weld. Black ash after welding was mainly the high temperature oxide of impurity elements. Under high cleaning speed, laser cleaning after welding slightly improved the elongation of the weld but had a small effect on porosity. When the laser was applied to the surface of aluminum alloy, the aluminum alloy underwent a melting and solidification process. A reinforced remelting layer appeared on the surface of the aluminum alloy [33]. Therefore, the elongation improved after post-cleaning of the welds.

## 5. Conclusions

In this paper, laser cleaning and MIG welding experiments were conducted on 6005A aluminum alloy for rail transportation. The improvement of laser cleaning on the performance of weld seams of large size aluminum alloy body parts and the regulation of welding defects were studied. This work provided new ideas for the application of aluminum alloy laser cleaning. The main conclusions are summarized as follows:

1. Laser cleaning can effectively reduce the surface oxygen content of aluminum alloy. The oxygen content decreases first and then increases with the increase of laser power and scanning frequency, with the increase of cleaning speed and decrease.
2. Laser cleaning produces craters on the surface of the sample and increases the surface roughness. Laser cleaning slightly improves the microhardness of the surface. At 0.5 m/min, the highest rise in hardness was 8.601%.
3. For the surfaces with oil and water, laser cleaning can effectively remove dirt and suppress the weld porosities. For the surfaces with oil and water, the porosity reduced from 28.672% and 2.702% to 0.091%, respectively. Black ash around the weld seam can be effectively removed by post-weld laser cleaning. Laser cleaning after welding slightly improves the elongation of the weld.

## Figures and Tables

**Table 1:** Main parameters of laser cleaning.

Parameters	Symbol	Value	Unit
Laser power	P	25, 50, 75, 100, 125, 150, 175, 200	W
Scanning frequency	Fs	25, 50, 75, 100, 125, 150, 175, 200	Hz
Cleaning speed	ν	0.5, 0.6, 0.7, 0.8, 0.9, 1.0, 1.1, 1.2	m/min
Scanning width	L	60	mm
Pulse frequency	Fp	130	kHz

**Table 2:** Main parameters of laser cleaning after welding.

Parameters	Symbol	Value	Unit
Laser power	P	150	W
Scanning frequency	Fs	125	Hz
Cleaning speed	ν	0.8	m/min
Scanning width	L	60	mm
Pulse frequency	Fp	130	kHz

## Data Availability

Not applicable.
